# “Sentinel” Circulating Tumor Cells Allow Early Diagnosis of Lung Cancer in Patients with Chronic Obstructive Pulmonary Disease

**DOI:** 10.1371/journal.pone.0111597

**Published:** 2014-10-31

**Authors:** Marius Ilie, Véronique Hofman, Elodie Long-Mira, Eric Selva, Jean-Michel Vignaud, Bernard Padovani, Jérôme Mouroux, Charles-Hugo Marquette, Paul Hofman

**Affiliations:** 1 Laboratory of Clinical and Experimental Pathology, Pasteur Hospital, Nice, France; 2 Human Biobank BB-0033-00025, Pasteur Hospital, Nice, France; 3 IRCAN Team 3, INSERM U1081/UMR CNRS 7284, Faculty of Medicine of Nice, University of Nice Sophia Antipolis, Nice, France; 4 Department of Pathology, Central Hospital, University of Nancy, Nancy, France; 5 Department of Radiology, Pasteur Hospital, Nice, France; 6 Department of Thoracic Surgery, Pasteur Hospital, Nice, France; 7 Department of Pulmonary Medicine, Pasteur Hospital, Nice, France; Cincinnati Children’s Hospital Medical Center, United States of America

## Abstract

Chronic obstructive pulmonary disease (COPD) is a risk factor for lung cancer. Migration of circulating tumor cells (CTCs) into the blood stream is an early event that occurs during carcinogenesis. We aimed to examine the presence of CTCs in complement to CT-scan in COPD patients without clinically detectable lung cancer as a first step to identify a new marker for early lung cancer diagnosis. The presence of CTCs was examined by an ISET filtration-enrichment technique, for 245 subjects without cancer, including 168 (68.6%) COPD patients, and 77 subjects without COPD (31.4%), including 42 control smokers and 35 non-smoking healthy individuals. CTCs were identified by cytomorphological analysis and characterized by studying their expression of epithelial and mesenchymal markers. COPD patients were monitored annually by low-dose spiral CT. CTCs were detected in 3% of COPD patients (5 out of 168 patients). The annual surveillance of the CTC-positive COPD patients by CT-scan screening detected lung nodules 1 to 4 years after CTC detection, leading to prompt surgical resection and histopathological diagnosis of early-stage lung cancer. Follow-up of the 5 patients by CT-scan and ISET 12 month after surgery showed no tumor recurrence. CTCs detected in COPD patients had a heterogeneous expression of epithelial and mesenchymal markers, which was similar to the corresponding lung tumor phenotype. No CTCs were detected in control smoking and non-smoking healthy individuals. CTCs can be detected in patients with COPD without clinically detectable lung cancer. Monitoring “sentinel” CTC-positive COPD patients may allow early diagnosis of lung cancer.

## Introduction

Despite recent progress into therapeutic strategies, the overall prognosis of lung cancer remains dismal, in particular at advanced stages [1], [2]. Only complete surgical resection of early-stage tumors improves the prognosis of non-small cell lung cancer (NSCLC) patients. One reason for the poor prognosis in NSCLC patients is the absence of routine, easy to perform and low cost methods that allow detection of asymptomatic early-stage tumors. The methods used previously for early diagnosis of lung carcinoma, including biological tests using blood samples, were not conclusive or needed to be confirmed on larger cohorts [3]–[5]. The early diagnosis of lung cancer is a critical public health issue since 94 million smokers have an elevated risk of developing the disease, which remains the leading cause of death in the US [6], [7]. The National Lung Screening Trial showed recently that low-dose CT screening is associated with a decrease in mortality from lung cancer of 20% [6]. However, this result was associated with 96.4% false positive results, since out of the 26,309 patients screened, 7,191 were found positive but only 649 were further revealed to have lung cancer. Furthermore, the total number of patients with lung cancer was 1,060, including 411 false negatives that were missed by CT screening. Thus, there is an urgent need to find new methods for early detection of lung cancer, particularly in “*at high-risk*” individuals.

Chronic obstructive pulmonary disease (COPD) and lung cancer share common pathophysiological pathways and epidemiological studies have shown that independently of the smoking status, the presence of COPD *per se* is a risk factor for NSCLC, even in early stage COPD [8]–[17].

Migration of circulating tumor cells (CTCs) into the blood stream seems to be an early event of human carcinogenesis as experimental data in animal models showed that tumors measuring less than 1 mm could be associated with the presence of CTCs in the blood stream [18]–[22]. Search for CTCs has been mainly performed, up to now, on patients with an established diagnosis of cancer, including patients with metastatic or localized cancer.

In this setting, we reasoned that the invasive character of lung cancer could be used as its “Achilles’ heel” and permit its early diagnosis through the sensitive and diagnostic detection of CTCs. Our results show for the first time that CTCs can be detected in a subpopulation of patients with COPD. CT-scan screening detected lung nodules 1 to 4 years after CTC isolation, leading to surgical resection and diagnosis of early-stage lung cancer. These results demonstrate the proof of concept of CTCs detection as an early indicator of invasive lung cancer in “*at risk*” patients.

## Patients and Methods

### Design and patients

This was a monocentric interventional, observational prospective study of patients with COPD. These patients were initially included in the experimental control group from a larger study (NCT00818558; Human Biobank, Nice, BB-0033-00025) of which the main purpose was the assessment of the presence and the frequency of CTCs in lung cancer patients undergoing surgery. The aim of this secondary study was to analyze the presence of CTCs in complement to CT-scan in COPD patients without clinically detectable lung cancer as a first step to identify a new marker for early lung cancer diagnosis. The patients received the necessary information concerning the study and written consent was obtained from each of them. The study was approved by the local Ethics Committee (04-APN-08, CHU de Nice, France).

245 subjects followed at the Pasteur Hospital (Department of Pulmonary Medicine, CHU of Nice, France) were enrolled in this study between June 2008 and April 2012. Among these subjects, 168 (68.6%) had COPD and 77 (31.4%) were without COPD, including 42 smokers without any detectable pathology (1<PY<30; mean age, 55±7 years; 22 men and 20 women) and 35 healthy non-smoking individuals (54±5 years; 20 men and 15 women). COPD patients did not have symptoms of clinically detectable lung cancer or other malignancies at the time of inclusion in the study. The main characteristics of the patients with COPD included in this study are described in Table 1. Patients with COPD had had no transbronchial and/or transparietal chest biopsies at least 15 days before bronchoscopy. Patients with COPD were first admitted to the hospital on the basis of physical findings, blood analysis, spiral computed tomography (CT) or fluorodeoxyglucose positron emission tomography. COPD patients with and without CTCs received low-dose spiral CT at baseline, and then annually as a part of the research study.

**Table 1:** Clinical and pathological characteristics of patients.

Variables	Overall n (%)
**COPD population**	**168 (100)**
**Age (years)**	
Mean ± SD	68.9±9.8
Range	35–89
**Gender**	
Male	104 (62)
Female	64 (38)
**Smoking status**	
Never smoked	20 (12)
Former smokers	89 (53)
Current smokers	59 (35)
**Cumulative smoking (PY)**	
Mean ± SD	56±32
Range	5–150
**COPD Severity**	
Mild	86 (51%)
Moderate	57 (34%)
Severe	25 (15%)
**Pulmonary function, mean (SD)**	
FEV1, L	2.8 (0.6)
FEV1, % predicted	83.8 (10.7)
FEV1/FVC, %	62.3 (5.2)

Abbreviations: PY: Packs-year, FEV1, forced expiratory volume in one second.

## Methods

### 

#### CTC detection

The Isolation by Size of Epithelial Tumor cells (ISET) technology (Rarecells Diagnostics, Paris, France) was carried out using previously described methodologies [23]. Briefly, the ISET method is a blood filtration-based approach, which enriches on a polycarbonate membrane cells larger than 8 microns. For ISET, 10 mL of peripheral blood was collected in buffered EDTA, maintained at room temperature and processed within 1 hour of collection. The membrane was cut into 2 parts containing respectively 6 spots for immunocytochemistry and 4 spots for May Grünwald Giemsa (MGG) staining for cytological analysis. Immunocytochemistry was performed as described previously, using double immunolabeling with a pan-cytokeratin antibody (mouse, clone KL-1, Immunotech-Beckman-Coulter, Villepinte, France), and an anti-vimentin (mouse, clone V9, Glostrup, Denmark) antibody applied to filters for 45 min at room temperature [23]. Using ISET, patients were considered positive for CTCs based on cytopathological analysis of the isolated cells and detection of cells with characteristic malignant features determined according to previously defined criteria [24]–[26].

Trial visits were defined as baseline, interim (around each 12 months from baseline), and final (around 5 years after baseline) visit. Data were collected from the physicians’ clinical notes at each visit, and along with CTCs data the information were transferred to a standard case report form.

#### Computed Tomography

CT thorax scans were acquired using a 64-slice GE Medical Systems Lightspeed Volume CT (GE Healthcare, Wisconsin, USA): detector array of 64×0.625 mm, FOV of 36 cm, table speed of 39.37 mm/s, rotation time of 0.5 s, 120 kVp, pitch of 0.987∶1, noise index of 17.36. After infusion of intravenous contrast media, the chest region was scanned during breath-hold at the end of inspiration in the supine position. Automated bolus tracking software was used to trigger image acquisition with a threshold of 100 HU in a region of interest placed over the thoracic aorta. Lung density and volume measurements were performed using the OsiriX digital analysis programme (OsiriX Imaging Software, v3.7.1, OsiriX Foundation, Geneva, Switzerland). Image segmentation software was used to segment lung parenchyma according to predetermined density thresholds. Lung attenuation threshold limits of −500 to −1024 HU were used to exclude soft tissue surrounding the lungs. Areas with attenuation values less than −910 HU were considered representative of poorly functioning emphysematous lung. One experimented radiologist, who was blinded to the CTC results and other data of patients, calculated measures of volume and density of each lung segment on the mediastinal window of CT images. The following CT parameters were calculated for each patient: total lung volume (TLV), the volume of normal lung parenchyma (values −500 HU to −910 HU), the volume of emphysematous lung (values less than −910 HU), and the mean lung density.

## Results

**Figure 1:**
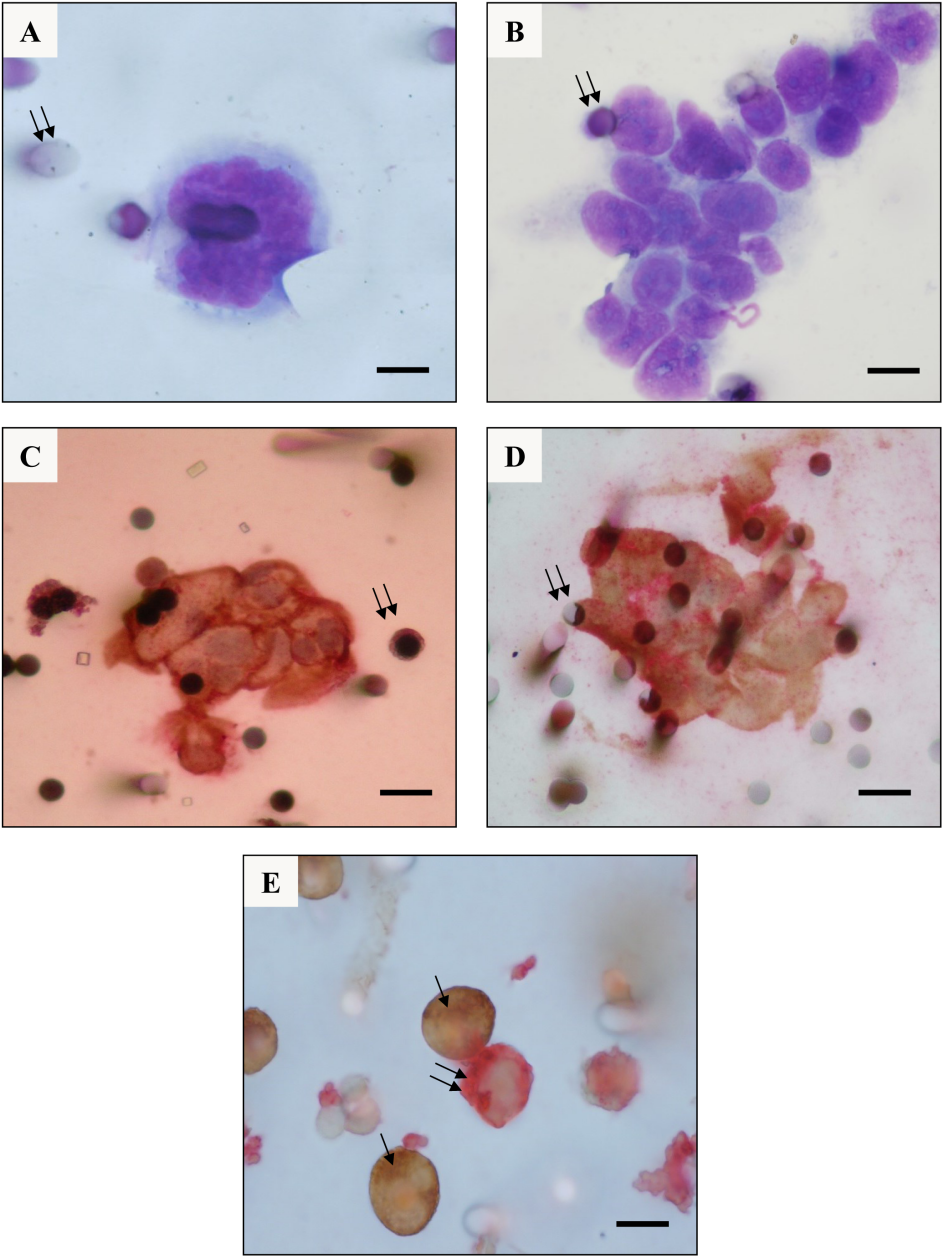
Cytomorphological and immunocytochemical analysis of Circulating Tumor Cells (CTCs) detected by the ISET technique in patients with COPD. (**A**) and (**B**) CTCs isolated by the ISET method and identified by MGG staining from Patient 1. (**A**) An isolated CTC with malignant cytomorphological features (Double arrows: pores of the filter). (**B**) A cluster (CTM) composed of 20 CTCs with malignant cytomorphological features (Original magnification×1000; bars: 8 µm; double arrows: pore containing a lymphocyte). (**C**) and (**D**) Immuno-stained CTMs observed in the blood filtered using the ISET method from Patient 2. (C) CTM strongly expressing the pan-cytokeratin antigen only (Double arrows: pore containing a lymphocyte). (**D**) CTM co-expressing pan-cytokeratin and vimentin antigens [Double arrows: pores of the filter, (Original magnification×400; bars: 16 µm; immuno-peroxidase staining with a pan-cytokeratin antibody (KL1), and an immuno-phosphatase staining with an anti-vimentin antibody)]. (**E**) A549 epithelial tumor cell line and K562 leukemic cell line having large vimentin aggregates were spiked in human blood, further filtered by ISET, and were used as positive controls for the double immunolabeling assays with KL1 (brown immuno-peroxidase staining, arrows) and with vimentin (reddish immuno-phosphatase staining, double arrows; Original magnification×1000; bars: 40 µm).

CTCs were detected in 3% (5 out of 168) of COPD patients based on blood filtration and the cytopathological analysis of the isolated cells. The detected cells had characteristic malignant cytopathological features (Fig. 1). CTCs revealed large nuclei, with scattered nuclear grooves, heterochromatin clumps, and a moderate amount of cytoplasm with a high nuclear/cytoplasmic ratio (Fig. 1). The five CTC-positive COPD patients were found to have between 19 and 67 isolated CTCs (Table 2). Moreover, these patients demonstrated occasional CTMs as follows: patient 1 had 1 CTM composed of 20 cells (Fig. 1); patient 2 had 3 CTMs composed of 5, 7 and 15 CTCs; patient 3 had 1 CTM with 16 CTCs; patient 4 had 1 CTM with 12 CTCs, and patient 5 had 1 CTM with 19 cells. Occasional clusters revealed tridimensional cohesive sheets of oval or polygonal CTCs showing nuclear atypia, moderate to prominent anisonucleosis, with frequent multiple nucleoli, and nuclear overlapping. The corresponding immunostained cells expressed mainly pan-cytokeratin alone (Fig. 1). However, a small number of CTCs strongly expressed vimentin with a weak associated cytokeratin expression (Fig. 1). The immunohisto/cytochemical analyses demonstrated a similar phenotype in CTCs and the corresponding lung tumors (*i.e*., strong positivity for KL1 and weak and focal positivity for vimentin; Fig. 1, Fig. S1).

**Table 2:** Clinical and pathological characteristics of CTC-positive COPD patients.

Patients	Sex	Age (years)	Smoking status (PY)	Year of COPD diagnosis	GOLD score	Year of CTC Detection (Study entry)	CTCs/CTM		Year of Lung cancer diagnosis	Lung Cancer size (cm)	Histology	Stage	Mutation tumor status	One-year follow-up after surgery
							CTCs	CTM						
**P1**	M	54	60	1998	3	2009	43	1	2012	1.9	Invasive papillary adenocarcinoma	IA	*KRAS* p.Gly12Cys	No recurrence
**P2**	F	48	45	1995	2	2009	67	3	2010	1.5	Invasive papillary adenocarcinoma	IA	*KRAS* p.Gly12Val	No recurrence
**P3**	M	47	35	1999	2	2008	32	1	2012	1.4	Invasive acinar adenocarcinoma	IA	*KRAS* p.Gly12Cys	No recurrence
**P4**	M	52	45	1994	3	2009	19	1	2013	2	Squamous cell carcinoma	IA	*STK11* (missense mutation)	No recurrence
**P5**	M	63	55	2001	3	2009	28	1	2013	1.5	Invasive acinar adenocarcinoma	IA	No mutation	No recurrence

The presence of CTC was significantly correlated to the severity of COPD (Table 1; Fisher’s exact test, *P*-value<0.001). We did not notice any relationship between the length of time with COPD and CTC detection [mean surveillance time, COPD(+CTC) group, 11 years *vs.* COPD(-CTC) group, 12 years].

The five COPD patients with CTCs detected by cytopathology analysis after blood-enrichment at baseline developed a lung cancer that was diagnosed at follow-up (Table 2). A CT-scan performed at the same time as the blood filtration confirmed the diagnosis of COPD but failed to show lung nodules. Furthermore, these patients had annual surveillance using low-dose spiral CT. After a mean follow-up of 3.2 years (range 1 to 4 years) the surveillance CT scan program revealed lung nodules. Surgery was performed on these 5 patients one month after nodule detection by CT scan. The pathological analysis demonstrated tumor nodules with a mean size of 1.7 cm in diameter. Four tumors were diagnosed as invasive adenocarcinoma and one patient developed a squamous cell carcinoma (Table 2). Cancer staging revealed stage IA lung cancer with no spread to lymph-nodes or distant metastases (pT1aN0M0) in all cases. The patients did not receive any further treatment. Tumor genotyping showed a *KRAS* mutation in codon 12 in three adenocarcinoma tumors, and a missense *STK11* gene mutation in the squamous cell carcinoma. One adenocarcinoma was wild type for *KRAS*, *EGFR* and *EML4-ALK* genomic alterations. Follow-up performed 16 months after surgery, including CT-scan and blood-filtration, showed no tumor recurrence and no detected CTCs.

Isolated cells with benign cytomorphological features were detected by ISET in 1.8% of COPD patients (3 out of 168 patients). None of these 3 patients and none of the other 160 patients with COPD, and with no pathological cells at baseline in the blood, were shown to develop a lung nodule, as demonstrated by a yearly CT-scan during the subsequent follow-up starting with the first blood filtration (mean follow-up time, 60 months). No CTCs were detected in the 42 control smoking individuals without a detectable pathology and in the 35 non-smoking healthy individuals (Fig. S2). These individuals have not developed lung nodules as demonstrated by the CT scan 5 years after the first inclusion in this research study.

## Discussion

Lung cancer is known to be a highly invasive cancer, with more than 75% of patients not eligible for surgery at diagnosis [27]. Because of its highly invasive character, it is the leading cause of cancer-related deaths worldwide [28]. In this field, the discovery of a diagnostic and non-invasive biomarker could be crucial to unroll the following steps of low-dose spiral CT-scan screening and early surgical intervention. Since the highly malignant behavior of lung cancer is bound to its invasive potential, we thought that the early detection of CTCs could complement CT-scan examination and help to reduce the false positive and negative results related to CT-scan screening. We thus targeted a population of 168 patients with COPD. COPD is the third leading cause of death in the U.S. and is projected to become the fourth leading cause of deaths worldwide by 2030, due to an increase in smoking [8], [14]. COPD is considered to be a pre-neoplastic condition of lung cancer and it has been calculated that, overall, 2.2% of COPD patients develop lung cancer per year [29]. Moreover, the progression of COPD increases the susceptibility to lung carcinogenesis by up to 4–6 fold, an observation that is thought to be due to shared mechanisms in both COPD and lung cancer [8], [30]. Thus, early diagnosis of COPD is important because smoking cessation early in COPD slows disease progression and decreases morbidity and mortality [31].

Several methods have been used to isolate and detect CTCs, with variable sensitivities and specificities [32]. However, we thought that, in the setting of an early diagnosis of lung cancer, a cytopathological and immunocytochemical diagnostic approach would be suitable to reveal “sentinel CTC/CTM” to be used in a combined approach as a reliable marker fostering CT-scan screening for early diagnosis of lung cancer [24]–[26].

We prospectively collected data on CTCs from 168 patients with COPD. Five out of 168 (3%) COPD patients had CTCs, which were isolated tumor cells or grouped into CTMs. CTCs revealed clear malignant features with large nuclei, frequent multiple nucleoli, anisonucleosis, and high nuclear/cytoplasmic ratio. The immunohisto/cytochemical analyses demonstrated a similar phenotype for the CTC and the corresponding lung tumors (i.e. strong positivity for KL1 and weak and focal positivity for vimentin).

The five cases reported here revealed a relevant high number of CTCs/CTMs 1 to 4 years before detection of a lung nodule by CT-scan. In particular, we found a long delay between the detection of CTC in Patient 3 and the detection of the tumor nodule by CT scan. Previous studies on cancer patients have established that disseminated tumor cells (DTC), which are mostly CTC that extravasated into tissues, remain in a dormant clinically undetected state for an extended period of time, before suddenly becoming metastatic [33]. While the mechanisms of tumor cell dormancy are still not clear, the condition is frequently observed in certain carcinomas [34]. Although our findings cannot generalize on such phenomenon in CTC-positive clinically dormant lung cancer patients, these data underline the importance of detecting CTC soon after the circulation of tumor cells in the blood has started as a “sentinel” to activate tumor nodule detection and treatment before CTC give rise to DTC and the subsequent risk of development of metastases.

Isolated cells with benign cytomorphological features were also detected by blood-filtration in 3 out of 168 (1.8%) COPD patients. However, none of these 3 patients and none of the 160 patients with COPD and with no CTC at baseline developed a lung nodule detectable by a yearly CT-scan during the subsequent follow-up (mean follow-up time: 48 months). This relevant finding is consistent with the clinical value of the classical cytopathological criteria used to identify malignant cells and applied to the detection of CTC [26].

In addition, no CTC were detected in 42 control smokers without a detectable pathology and in 35 non-smoking healthy individuals. Overall, approximately 620 subjects without cancer have been studied by ISET by different groups and shown to be without CTC in their blood [23]–[26], [35]. Taken together, these data strongly suggest that the cytopathological and immunocytopathological detection of CTC in patients at a high risk of developing cancer may play a “sentinel” role triggering follow-up programs aimed at the early detection of invasive cancers. With lung cancer being considered among the most deadly type of cancer, with approximately 87% of patients dying at 5 years after diagnosis, these data clearly raise the hope of reducing the rate of mortality due to lung cancer, through its early detection.

Consistently, several studies have shown that the detection of CTCs in early and/or metastatic diseases correlated with an unfavorable clinical outcome [23], [36]. Hence, when CTC are present in patients with a presumably localized tumor, they could contribute to disease progression [23]. In this regard, we demonstrated previously that detection of CTCs in lung cancer patients, including those with localized “early stage” lung cancer, strongly correlated with worse overall survival [37].

Despite the fact that several models and animal studies have suggested that the presence of CTCs in patients’ blood is an early event in patients developing cancer, this is the first study showing that CTCs may be detected in the absence of a cancer nodule detectable by CT scan and be a hallmark of a developing invasive cancer. While conventional hypotheses assume that invasion and metastases are late events, convergent results have led to the notion that invasion can occur very early and is sometimes clinically dormant [21], [38], [39]. In this regard, tumor-induced neovascularization occurs in parallel with transition to invasion and provides a vascular entry portal for dissemination, which may precede primary tumor outgrowth by many years [39]. It is noteworthy that tumor cell spread may start years before diagnosis and the probability of tumor cells spreading from small cancers has been reported to be high [38], [40]. From model systems, it has been estimated that around 1.10^6^ tumor cells per gram of tumor tissue can enter daily into the bloodstream [20]. Recent studies in animals have demonstrated that tumor cell circulation starts very early, at the “*in situ* carcinoma” stage [22]. Another model suggests that the cellular determinants for invasion may be present even before angiogenesis and that subsequent development of new vessels can provide the final requirement for tumor cell spread [41]. Finally, recent molecular studies have shown that the capacity to metastasize may be pre-ordained by the spectrum of mutations acquired very early in tumorigenesis, suggesting that the detection of CTCs in pre-neoplastic lesions may be a useful tool for early-diagnosis of cancer [42]. However, oncogenic *KRAS* has differing effects – in some cases, it induces hyperproliferation, whereas in others it leads to oncogene induced senescence [43]. In particular, endogenous oncogenic *KRAS* (*KRAS^G12V^*) was shown to trigger senescence in pre-malignant stages of lung and pancreatic tumors [44], [45]. Interestingly, tumors with high levels of oncogene expression progress to full-blown carcinomas only when senescence is cancelled by the genetic deletion of Cdkn2a or Trp53 [44], [45]. This phenomenon may explain the relative late development of lung tumors in the CTC-positive COPD patients.

Our study shows that diagnostic CTC detection and imaging can be used together to improve early lung cancer detection. In this setting, among the tools for early lung cancer detection, [^18^F] fluorodeoxyglucose (FDG) positron emission tomography (PET)/computed tomography is certainly the most used and sensitive method in the clinical practice and recent studies have started to compare the efficiency of FDG-PET and CTC detection in monitoring breast carcinoma patients [46], [47]. Recently, a novel radiotracer was developed to image glycogen metabolism in tumors by PET. The authors demonstrated that glycogen levels, but not [^18^F]-FDG uptake, increase proportionally with cell density and G1-G0 arrest, during the non-proliferative state of cancer cells, with potential application in the assessment of the detection of tumor quiescence [48].

In conclusion, this study shows for the first time that a sensitive and diagnostic CTC detection approach can find CTCs in patients “*at risk*” of developing lung cancer without a detectable nodule by CT scan. A small fraction of COPD patients were found to have CTCs 1 to 4 years before identification of a lung nodule by imaging. In these 5 patients the lung cancer was diagnosed at an early stage (IA) allowing prompt surgical resection; they were then shown to be without tumor recurrence and without CTCs 16 months after surgery.

Larger studies are needed to independently validate the potential of diagnostic identification of CTCs as a reliable tool fostering targeted, intensive surveillance by CT-scan for early diagnosis of lung cancer in “*at risk*” patients. Moreover, follow-up studies are needed to clarify the predictive impact of these tests, including studies targeting COPD patients with a nodule measuring less than 10 mm detected by CT and to assess the predictive value of CTC detection for early invasive cancer screening in COPD patients.

## Supporting Information

Figure S1 **Immunostaining for KL1 and vimentin in lung tumors from patients with CTC-positive COPD.** Example of the lung adenocarcinoma from patient 2. **(A)** Strong staining with the KL1 pan-cytokeratin antibody in all tumor cells**. (B)** Weak expression of vimentin in a majority of tumor cells (black arrows). Yellow arrowheads point the strong vimentin expression in the tumor stroma. **(C)** Focal intense expression in some tumor cells (black arrows). Yellow arrowheads point the strong vimentin expression in the tumor stroma. (Original magnification×200).
(TIF)

Figure S2 **Lack of CTCs in (A) control non-smoking healthy individuals, and in (B) smoking subjects, as demonstrated by cytomomorphology on ISET filters.** Arrows point white blood cells. (Original magnification×200).
(TIF)
