# Supplementary figures and images for: “Sentinel” Circulating Tumor Cells Allow Early Diagnosis of Lung Cancer in Patients with Chronic Obstructive Pulmonary Disease
Source: PLoS One. 2014 Oct 31;9(10):e111597. doi: 10.1371/journal.pone.0111597 (PMC4216113; doi:10.1371/journal.pone.0111597)

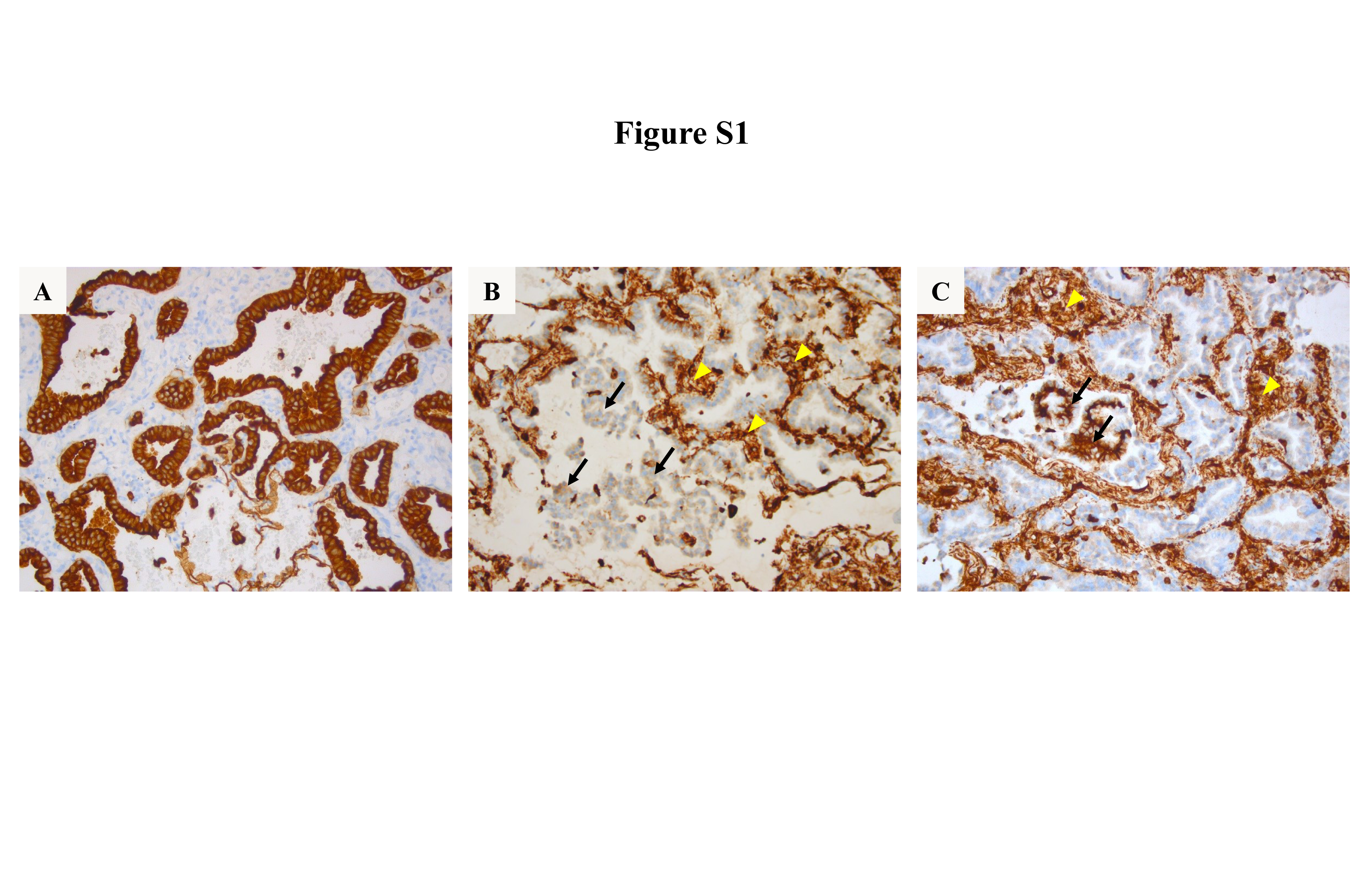

Supplement: Figure S1 — Immunostaining for KL1 and vimentin in lung tumors from patients with CTC-positive COPD. Example of the lung adenocarcinoma from patient 2. (A) Strong staining with the KL1 pan-cytokeratin antibody in all tumor cells. (B) Weak expression of vimentin in a majority of tumor cells (black arrows). Yellow arrowheads point the strong vimentin expression in the tumor stroma. (C) Focal intense expression in some tumor cells (black arrows). Yellow arrowheads point the strong vimentin expression in the tumor stroma. (Original magnification×200). (TIF) [file pone.0111597.s001.tif]

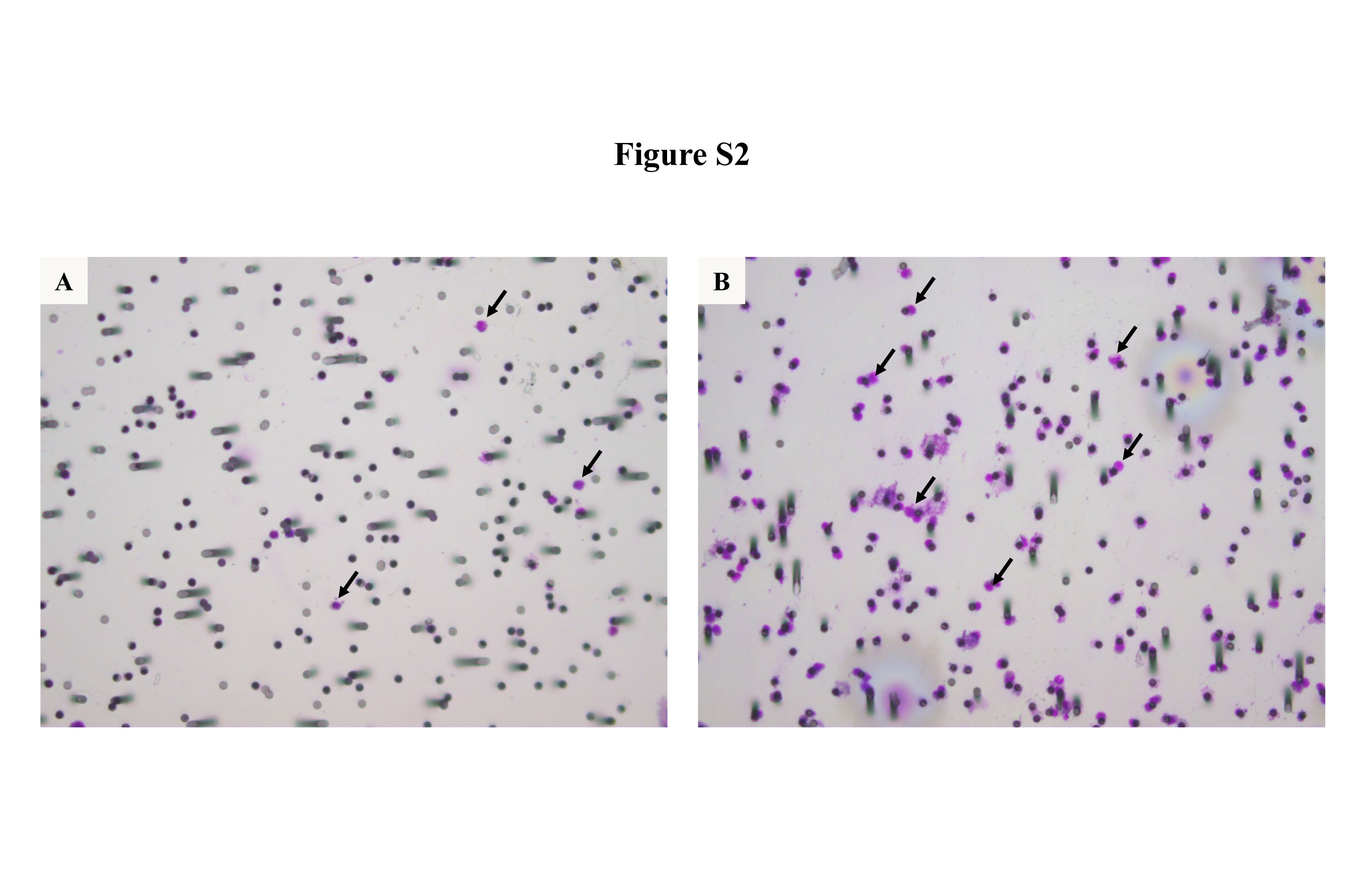

Supplement: Figure S2 — Lack of CTCs in (A) control non-smoking healthy individuals, and in (B) smoking subjects, as demonstrated by cytomomorphology on ISET filters. Arrows point white blood cells. (Original magnification×200). (TIF) [file pone.0111597.s002.tif]
